# Cardiovascular response to altered gravity in healthy adults: Insight from graded tilt testing

**DOI:** 10.14814/phy2.70782

**Published:** 2026-02-19

**Authors:** Adrien Robin, Richard S. Whittle, Ana Diaz‐Artiles

**Affiliations:** ^1^ Aerospace and Extreme Environment Nursing Program, College of Nursing Texas A&M University Bryan Texas USA; ^2^ Department of Aerospace Engineering Texas A&M University College Station Texas USA; ^3^ Department of Mechanical and Aerospace Engineering University of California‐Davis Davis California USA; ^4^ Department of Medical Physiology Texas A&M University College Station Texas USA

**Keywords:** altered gravity, dose–response, hemodynamics, sex differences, vascular ultrasound

## Abstract

Microgravity exposure during spaceflight induces a thoracocephalic fluid shift that affects the cardiovascular system both during flight and after return to Earth. As the proportion of female astronauts increases, it is essential to understand how altered gravity impacts cardiovascular function across sexes. In this study, we examined sex differences in central hemodynamics, vascular morphology of the common carotid artery and internal jugular vein (IJV), and IJV pressure during graded head‐up to head‐down tilt (+45° to −45° in 15° increments) in healthy participants (12 female and 12 male adults). A strong gravitational dependence on almost all variables was observed, except for oxygen consumption. Only a few variables showed significant sex differences, and these include cardiac output, total peripheral resistance, rate pressure product, oxygen consumption, and sympathovagal balance (LF/HF ratio). Overall, hemodynamic, vascular morphology, and IJV pressure responses to tilt were largely similar between sexes. The additional female gravitational dose–response curves augment our previous, male‐only database of cardiovascular responses to tilt. Together, these results provide a unique and more comprehensive normative baseline to support the development of spaceflight countermeasures as well as other terrestrial clinical applications, such as surgery in Trendelenburg or prone positioning.

## 1 INTRODUCTION

Spaceflight induces a thoraco‐cephalic fluid shift and redistribution that affect the cardiovascular system both during flight and after return to Earth due to changes in gravitational forces. Sex‐related differences have been observed in cardiovascular adaptation to spaceflight or microgravity simulations (Hughes‐Fulford et al., 2024), women being more susceptible to post‐flight orthostatic intolerance, more pronounced hypovolemia, and lower vasoconstrictive reserve and baroreflex impairment (Evans et al., 2018; Mark et al., 2014). Further, multiple studies have noted sex‐dependent differences in hemodynamic function, autonomic response, and in extracranial neck vascular hemodynamics, including blood pressure control, carotid artery flow and diameter, vascular geometry, and jugular vein distension (Arzeno et al., 2013; Choudhry et al., 2016; Krejza et al., 2006; Patterson et al., 2022; Scheel et al., 2000). Where the venous thromboembolism events (VTE) concern is considered, it should also be noted that the case report of a venous thrombosis onboard the International Space Station (ISS) was detected in a female crewmember (Marshall‐Goebel et al., 2019). Given that the spaceflight environment also promotes hypercoagulability and endothelial dysfunction, it is imperative to investigate whether similar hemodynamic responses occur in females and how they might influence thrombotic risk during spaceflight.

As future spaceflight will see a higher proportion of female crewmembers, it is important to consider the effect of sex when examining cardiovascular function, and as commercial spaceflight expands (e.g., recent Inspiration 4 and Polaris Down missions), civilian crewmembers may face increased health risks. Unlike the rigorously selected and medically screened professional astronaut's corps, civilians may travel to space with lower medical clearance standards, potentially including individuals with multiple preexisting conditions or undiagnosed, asymptomatic cardiovascular issues (Jennings et al., 2006; Stepanek et al., 2019).

To facilitate the study of cardiovascular responses in controlled environments, dry‐immersion (Horeau et al., 2024; Robin et al., 2020, 2023; Robin, Navasiolava, et al., 2022; Twomey et al., 2021) and head‐down tilt (HDT) bedrest (Pavy‐Le Traon et al., 2007; Robin, Wang, et al., 2022) are widely accepted terrestrial models for simulating fluid redistributions and subsequent effects during microgravity exposure. In contrast to prolonged HDT or dry‐immersion protocols lasting hours to days, which better reproduce sustained fluid shifts and deconditioning, the present graded tilt protocol was designed to characterize acute responses across a range of orthostatic stresses, providing mechanistic insight that can inform the interpretation and design of longer‐duration analog and spaceflight studies. Prior research utilizing HDT has predominantly focused on male subjects, leaving a gap in understanding how these gravitational changes influence cardiovascular physiology in females, and this, despite known sex‐based differences in cardiovascular anatomy and autonomic regulation. Most tilt studies evaluate supine posture only, yet body orientation changes the anteroposterior (*G*_x_) component of gravity. Comparing supine and prone tilts reverses the *G*_x_ vector while keeping tilt magnitude constant, isolating the contribution of tissue‐weight distribution to venous return and regional perfusion (Buckey et al., 2018; Tugrul et al., 2004). We therefore included both orientations to test posture‐dependent mechanisms in gravitational hemodynamics. Thus, in this study, we extend our previous work (Whittle et al., 2022; Whittle & Diaz‐Artiles, 2023) on graded tilt in prone and supine position by incorporating 12 additional female subjects. We keep the methodology and protocol the same as in the original experiment but add Sex as an additional factor into our gravitational dose–response models.

This study aims to fill the existing knowledge gap by examining the acute cardiovascular and autonomic responses to a wide range of gravitational levels by using a head‐up and head‐down tilt paradigm in female and male participants. By systematically varying the tilt angle and assessing hemodynamic parameters, we seek to characterize the dose–response relationship between changes in the gravitational vector and cardiovascular function in both sexes. We hypothesized that graded tilt in supine and prone positions would elicit angle‐dependent changes in central hemodynamics, autonomic indices, and internal jugular vein characteristics, and that these dose–response profiles would differ between women and men. This research provides valuable insights into sex‐specific cardiovascular adaptations to altered gravitational environments, informing both clinical practice on Earth and health risk assessment for female astronauts during spaceflight.

## 2 MATERIALS AND METHODS

### 2.1 Subjects and study approval

Subjects recruitment and inclusion criteria have been fully described in previous studies (Whittle et al., 2022; Whittle & Diaz‐Artiles, 2023). Here, in addition to the 12 male subjects, we further recruited 12 healthy, recreationally active female subjects selected in order to match the age range and body mass index (BMI) of the male subjects. Exclusion criteria included current use of any cardiac, blood pressure, muscle relaxant, anticoagulant, or stimulant medications, thyroid disease, chronic cardiovascular pathologies, extreme obesity, and history of hypertension, with the addition of pregnancy as an exclusion for female participants. Participants were excluded if they reported regular use of stimulant or other vasoactive/psychoactive medications expected to affect cardiovascular regulation, and they were instructed to abstain from caffeine, alcohol, and tobacco consumption before each testing session. Subject characteristics (mean ± SD), including blood pressure at screening, are shown in Table 1. The data also present a comparison with the male subjects as assessed by a two‐sample *t*‐test. All procedures performed in the study were in accordance with the 1964 Helsinki Declaration and its later amendments. The study protocol was approved by the Texas A&M Human Research Protection Program with Institutional Review Board number IRB2020‐0724F, and all participants gave written informed consent.

**TABLE 1:** Characteristics of the 12 recreationally active female subjects who participated in the study.

Characteristic	Male	Female	p
n	12	12	—
Race	W (8), B (1), A (3)	W (6), A (6)	—
Age (years)	26.8 ± 2.9	27.9 ± 4.4	0.479
Height (cm)	179.0 ± 8.3	159.4 ± 6.9	<0.001***
Weight (kg)	84.7 ± 18.7	61.3 ± 15.9	0.003**
BMI (kg/m^2^)	26.3 ± 4.9	24.1 ± 6.4	0.344
SBP (mmHg)	129.5 ± 14.5	120.3 ± 18.7	0.192
DBP (mmHg)	82.3 ± 6.5	80.8 ± 13.1	0.722

*Note*: Characteristics are presented alongside the 12 male subjects from our previous study (Whittle et al., 2022) and were recorded during baseline session prior to testing sessions. Data are reported as mean ± SD where appropriate. Race categories: W, White; B, Black or African American; A, Asian. The final column, *p*, shows where the values are significantly different from the male subjects as assessed by a two‐sample *t*‐test: ***p < 0.001, **p < 0.01, *p < 0.05.

Abbreviations: BMI, body mass index; DBP, diastolic blood pressure; SBP, systolic blood pressure.

### 2.2 Experimental design and testing protocol

The experimental design and testing protocol was identical to the procedure described by Whittle et al. (2022)., Briefly, participants completed two experimental sessions within a 2‐week period, during which an initial seated baseline measurement was performed on a standard chair, then they were assisted onto the tilt table (World Triathlon Corporation, Tampa Bay, FL) and brought directly to +45° head‐up tilt (HUT) before incrementally moving to 45° head‐down tilt (HDT) in 15° increments. Each participant completed one session in the supine (face‐up) posture and another in the prone (face‐down) posture, with the order counterbalanced between subjects. In the prone position, participants rested with their forehead supported on a thin cushion designed to (1) offload the weight of the head and minimize neck muscle strain, (2) maintain cervical spine alignment comparable to the supine posture, and (3) allow unobstructed breathing, with the mouth and nose slightly offset from the table surface. At each tilt angle, participants rested for approximately 12 min: 5 min of acclimation followed by 7 min of data acquisition, during which both continuous and discrete hemodynamic and autonomic variables were measured. This protocol was applied across all tilt angles, as well as during a seated baseline session preceding the main experiments. One subject was unable to complete the 45° HDT condition in both the supine and prone positions due to discomfort; an additional subject was unable to complete the 45° HDT condition in the prone position. Both subjects were returned to a HUT position and experienced no lasting symptoms. The remainder of their data are included in the results. All other subjects completed the full protocol and experienced no adverse effects.

### 2.3 Hemodynamics measurements

The dependent variables collected were identical to those described in Whittle et al. (2022). Briefly, hemodynamic measurements were recorded using an Innocor gas rebreathing device (Cosmed: The Metabolic Company, Rome, Italy) for heart rate (HR; beats per minute), stroke volume (SV; milliliters), cardiac output (CO; liters per minute) and oxygen consumption (V̇O_2_; liters per minute), and a Finapres NOVA (Finapres Medical Systems B.V., Enschede, the Netherlands) for systolic blood pressure (SBP; millimeters of mercury) and diastolic blood pressure (DBP; millimeters of mercury), rate pressure product (RPP; millimeters of mercury per minute; calculated as HR × SBP averaged over the analysis window). Total peripheral resistance was calculated as the ratio between mean arterial pressure (from the Finapres) and CO (from the Innocor). Finapres data were collected continuously throughout the protocol. Pressure was corrected to heart level with a hydrostatic height sensor, and calibrated at each tilt angle with a brachial blood pressure measurement.

Autonomic analysis was performed from measurements of heart rate variability (HRV) and baroreflex sensitivity as described in Whittle et al. (2022). Three time‐domain and three frequency‐domain indices were considered. The three time‐domain indices were: (1) the standard deviation of the NN intervals (SDNN); (2) the root mean square of direct differences of the NN interval (RMSDD); and (3) heart rate variability triangular index (HRVTi). As a time‐dependent measure of autonomic function, baroreflex sensitivity (BRS) was also included in this set of metrics. The three frequency‐domain indices were: (1) spectral power density in the low frequency (0.04–0.15 Hz) band (LF); (2) spectral power density in the high frequency (0.15–0.4 Hz) band (HF); and (3) the ratio between low frequency and high frequency power spectral densities (LF/HF). The LF and HF are shown in both absolute units (ms^2^) and normalized units (LFNorm and HFNorm), which represent relative contributions of each power component in proportion to the total power minus the very low frequency (VLF, 0.0033–0.04 Hz) component. HFNorm was used as an index of cardiac parasympathetic modulation, whereas LFNorm and the LF/HF ratio were used as conventional indices of overall sympathovagal modulation. We recognize that LF power and LF/HF are influenced by both sympathetic and parasympathetic inputs. in the present study these indices are therefore interpreted as markers of autonomic modulation rather than pure sympathetic drive.

### 2.4 Carotid and jugular vascular imaging

B‐mode vascular images were obtained using a handheld point‐of‐care ultrasound scanner (males: VScan Extend, GE Healthcare, Chicago, IL; females: Butterfly iQ+, Butterfly Network Inc., Burlington, MA). For all participants, cross‐sectional areas of the common carotid artery (A_CCA_) and internal jugular vein (A_IJV_) were imaged in short axis with a linear array using vascular presets as described in Whittle & Diaz‐Artiles (2023). All areas were manually traced onto the images by a trained operator. In all participants, internal jugular vein pressure (IJVP) was obtained with a non‐invasive compression sonography device (VeinPress, VeinPress GmbH, Münsingen, Switzerland) attached to the probe head of the ultrasound, following the same methodology as performed previously in flight (Lee et al., 2020; Martin et al., 2016) and on ground (Hearon et al., 2023; Whittle et al., 2025; Whittle & Diaz‐Artiles, 2023). Pressure values were obtained in duplicate (and then averaged) by manually compressing the IJV until the vessel walls touching point was reached.

### 2.5 Statistical analysis

In order to generate the dose–response curves while including the effect of Sex, we use a similar procedure to the one fully described in previous work (Whittle et al., 2022; Whittle & Diaz‐Artiles, 2023), with an additional fixed effect of Sex and associated interaction effects. We constructed dose–response curves using linear mixed‐effects models (LMMs), generalized linear mixed‐effects models (GLMMs, gamma distribution/log link), and generalized additive mixed‐effects models (GAMMs) for the systemic hemodynamic, autonomic, and cephalad measurements, respectively. For the LMMs and GLMMs, the linear predictor took the form:

(1) $$\begin{array}{c} \eta_{\text{ijkl}}=\beta_{0}+\beta_{1}\sin\left({\text{Angle}}_{j}\right)+\beta_{2}\left({\mathrm{Sex}}_{k}\right)+\beta_{3}\left(\text{Positio}n_{l}\right)\\ \;+\beta_{4}\left(\sin\left({\text{Angle}}_{j}\right)\times {\mathrm{Sex}}_{k}\right)+\beta_{5}\left(\sin\left({\text{Angle}}_{j}\right)\times \text{Positio}n_{l}\right)\\ \;+\beta_{6}\left({\mathrm{Sex}}_{k}\times \text{Positio}n_{l}\right)+\beta_{7}\left(\sin\left({\text{Angle}}_{j}\right)\times {\mathrm{Sex}}_{k}\times \text{Positio}n_{l}\right)+\gamma_{i}+\varepsilon_{\mathrm{ij}} \end{array}$$

where for each dependent variable, the linear predictor *η*_*ijkl*_ for subject *i* (*i* = 1: 24) is described by the tilt Angle (*j* = 1: 7, from 45° HUT to 45° HDT), the Sex of the subject (*k* = 1: 2, male or female), Position (l = 1: 2, supine or prone), the fixed effects *β* (where *β*_*0*_ represents the intercept), the random intercept *γ*_*i*_ (associated with each subject and the within‐subjects design), and the residual error *ε*_*ijkl*_. Dose–response curves are shown as mean and 95% confidence band. If the main effect of a factor and any interactions involving that factor were not significant, that factor was removed. Interaction effects were only included if statistically significant.

For the cephalad measurements, the dose–response of the GAMM was given by Equation 2, where separate smoothed splines were used for each significant parametric effect including the Side (*m* = 1: 2, right or left):

(2) $$\begin{array}{c} Y_{\text{ijklm}}=\beta_{0}+\beta_{1}\left({\mathrm{Sex}}_{k}\right)+\beta_{2}\left({\text{Position}}_{l}\right)\\ \;\;\;+\beta_{3}\left({\text{Side}}_{m}\right)+f_{\mathrm{klm}}\left(\sin\left({\text{Angle}}_{j}\right)\right)+\gamma_{i}+\varepsilon_{\text{ijklm}} \end{array}$$

Diagnostics were assessed using the same procedure as described in Whittle et al. (2022). All statistical analyses were completed using R version 4.2.2 (R Core Team, 2023) with LMMs and GLMMs fit using the lme4 (Bates et al., 2024) and glmmTMB (Brooks et al., 2017) packages. GAMMs were fit using the mgcv package (Wood, 2011). Diagnostics were assessed using the lmerTest (Kuznetsova et al., 2017) and DHARMa (Hartig et al., 2021) packages. Significance level was set at *α* = 0.05 (two‐sided).

Data are presented as mean ± SE in figures and text (unless indicated otherwise), as we aim to emphasize the precision of the mean responses and the shape of the tilt‐induced profiles, rather than the variability among individual observations.

## 3 RESULTS

### 3.1 Experimental data

**FIGURE 1:**
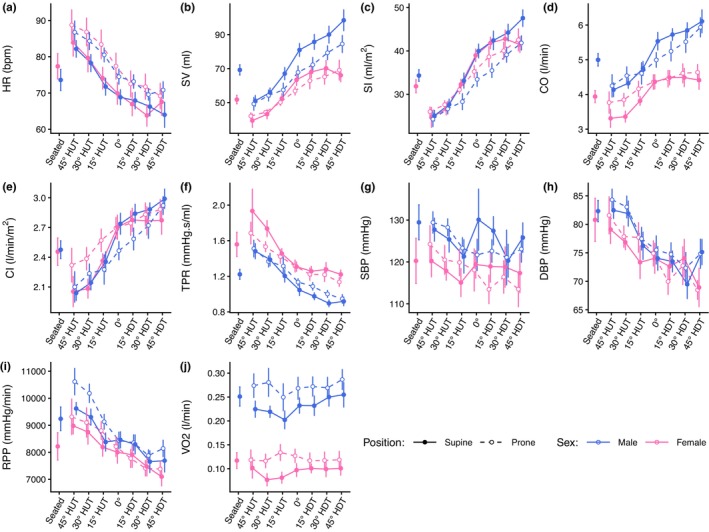
Hemodynamic variables as a function of tilt angle in supine and prone positions in women and men. (a) HR, heart rate; (b) SV, stroke volume; (c) SI, stroke volume index; (d) CO, cardiac output; (e) CI, cardiac index; (f) TPR, total peripheral resistance; (g) SBP, systolic blood pressure; (h) DBP, diastolic blood pressure; (i) RPP, rate pressure product; (j) V̇O_2_, oxygen consumption. Measurements were collected at a seated baseline, 45° head‐up tilt (HUT), 30° HUT, 15° HUT, 0°, 15° head‐down tilt (HDT), 30° HDT, and 45° HDT. Data were collected on 12 male and 12 female participants and are presented as means ± SE at each tilt angle.

Figure 1 shows the evolution of hemodynamic parameters (mean ± SE) as a function of tilt angle (including the seated baseline). Table 2 reports the results of the LMM analyses. Results for the female subjects follow the same trend as results from the male subjects. All variables, with the exception of V̇O_2_, show a linear effect of tilt angle. For SBP, a small effect of tilt angle appears present; however, it remains largely controlled across the tilt range measured and did not differ between sexes. There is a significant main effect of Sex for RPP (*p* = 0.042) and V̇O_2_ (*p* < 0.001). On average, males have an RPP that is 1020 ± 490 mmHg/min (effect size *t*_52_ = 2.082) higher than females, and a V̇O_2_ that is 0.143 ± 0.025 L/min (*t*_61_ = 5.797) higher than females. CO does not have a significant main effect of Sex (*p* = 0.363), but it does have a significant interaction effect between Angle and Sex (*p* = 0.011), such that CO increases 0.11 ± 0.04 L/min/15° faster in males than in females (*t*_302_ = 2.552), and this interaction effect is not observed when CO and SV are normalized to body surface area (SI, stroke volume Index; CI, Cardiac Index). Similarly, TPR does not have a significant main effect of Sex (*p* = 0.189), but it presents a significant interaction effect between Sex and Position (*p* = 0.037), with TPR in males being lower than in females in the supine position. Contrary to our results previously published (Whittle et al., 2022), when female subjects are included, we find no effect of Position on SV (higher in supine with respect to prone by 0.12 ± 3.39 mL, *t*_298_ = 0.036, *p* = 0.971). We further find no effect of Position on RPP (lower in supine with respect to prone by 530 ± 310 mmHg/min, *t*_302_ = −1.714, *p* = 0.088).

**TABLE 2:** Statistical results of the linear mixed model and generalized linear mixed model analysis incorporating sex differences.

	Significance p						
	Angle	Sex	Position	Angle × sex	Angle × position	Sex × position	Angle × sex × position
Hemodynamic measurements							
HR	<0.001***	0.397	<0.001***	0.296	0.160	0.455	0.492
SV	<0.001***	0.283	0.971	0.080	0.559	0.771	0.198
SI	<0.001***	0.657	0.865	0.117	0.388	0.628	0.363
CO	<0.001***	0.363	0.011*	0.011*	0.102	0.184	0.889
CI	<0.001***	0.693	0.004**	0.191	0.056	0.097	0.859
TPR	<0.001***	0.189	0.008**	0.685	0.086	0.037*	0.410
SBP	0.012*	0.358	0.120	0.646	0.073	0.474	0.538
DBP	<0.001***	0.469	0.186	0.837	0.228	0.601	0.406
RPP	<0.001***	0.042*	0.088	0.242	0.243	0.298	0.663
V̇O_2_	0.821	<0.001***	0.018*	0.602	0.385	0.367	0.754
Time‐domain autonomic indices							
SDNN	<0.001***	0.908	0.017*	0.894	0.229	0.456	0.718
RMSDD	<0.001***	0.876	0.051	0.218	0.646	0.754	0.862
HRVTi	<0.001***	0.743	0.003**	0.408	0.042*	0.275	0.556
BRS	<0.001***	0.197	0.009**	0.115	0.346	0.090	0.296
Frequency‐Domain Autonomic Indices							
LF	0.003**	0.439	0.004**	0.783	0.564	0.404	0.737
HF	<0.001***	0.904	0.030*	0.305	0.117	0.336	0.456
LFNorm	<0.001***	0.566	0.431	0.107	0.574	0.825	0.588
HFNorm	<0.001***	0.565	0.431	0.107	0.574	0.824	0.589
LF/HF	<0.001***	0.883	0.739	0.021*	0.345	0.412	0.078

*Note*: Fixed factors included angle, sex, position, and their interactions. Subjects were included as random factors. See text for abbreviations and model details.

*** *p* < 0.001.

** *p* < 0.01.

* *p* < 0.05.

**FIGURE 2:**
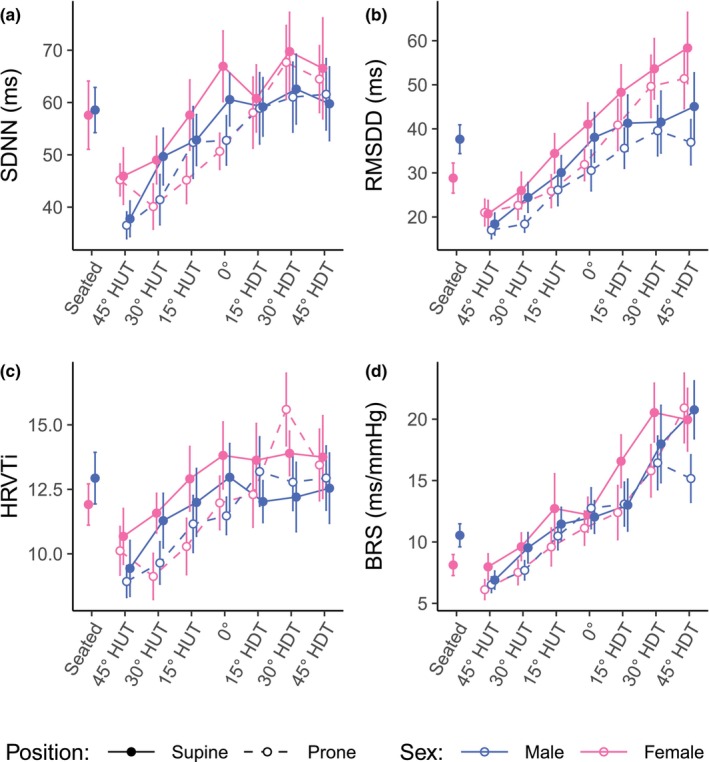
Time‐domain autonomic indices as a function of tilt angle in supine and prone positions, in women and men. (a) SDNN, standard deviation of NN intervals (normalized RR intervals); (b) RMSDD, root mean square of direct differences of NN intervals; (c) HRVTi, heart rate variability triangular index; (d) BRS, baroreceptor sensitivity. Measurements were collected at a seated baseline, 45° head‐up tilt (HUT), 30° HUT, 15° HUT, 0°, 15° head‐down tilt (HDT), 30° HDT, and 45° HDT. Data were collected on 12 male and 12 female participants. Data are presented as means ± SE at each tilt angle.

Figure 2 shows the evolution of time‐domain autonomic indices (mean ± SE) as a function of tilt angle (including the seated baseline). Table 2 reports the results of the GLMM analyses. In none of the variables do we find a significant main effect of Sex, or any significant interactions involving Sex. Further, contrary to our results previously published (Whittle et al., 2022), when the female subjects are considered, we find significant main effects of Position for SDNN, HRVTi, and BRS, but no significant main effect of Position for RMSDD (*z* = 1.95, *p* = 0.051). For SDNN, the index is, on average, 1.23 times higher in the supine position than in the prone position (*z* = 2.39, *p* = 0.017). BRS is 1.39 times higher in the supine position than in the prone position (*z* = 2.62, *p* = 0.009). For HRVTi, there is both a significant main effect of Position (*z* = 2.97, *p* = 0.003) and a significant interaction between Angle and Position (*z* = −2.04, *p* = 0.042).

**FIGURE 3:**
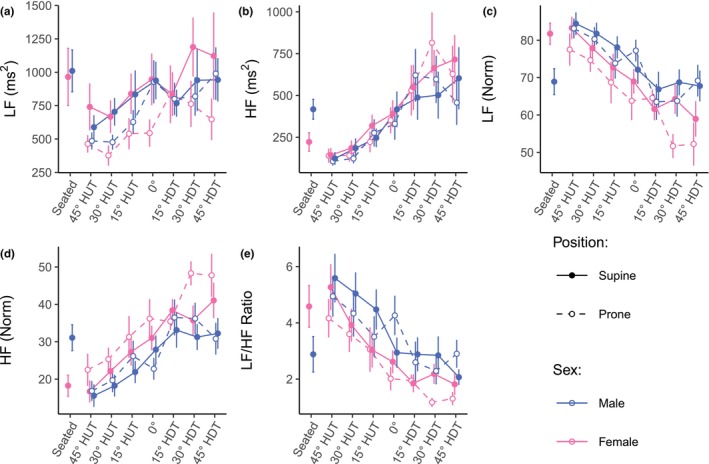
Frequency‐domain autonomic indices as a function of tilt angle in supine and prone positions, in women and men. (a) LF, power density in the low frequency range (0.04– 0.15 Hz); (b) HF, power density in the high frequency range (0.15– 0.4 Hz); (c) LFNorm, LF (normalized units); (d) HFNorm, HF (normalized units); (e) LF/HF Ratio, ratio of low to high power densities. Measurements were collected at a seated baseline, 45° head‐up tilt (HUT), 30° HUT, 15° HUT, 0°, 15° head‐down tilt (HDT), 30° HDT, and 45° HDT. Data were collected on 12 male and 12 female participants. Data are presented as means ± SE at each tilt angle.

Figure 3 shows the evolution of the frequency‐domain autonomic indices (mean ± SE) as a function of tilt angle (including the seated baseline). Table 2 reports the results of the LMM and GLMM analyses. With the inclusion of the female subjects, the normalized low‐ and high frequency gravitational dose–response is similar to the dose–response with only male subjects. However, we find a significant interaction effect between Angle and Sex in the LF/HF ratio with the decrease in LF/HF ratio being 1.10 times lower in males with respect to females (*z* = 0.2.306, *p* = 0.021). Further, with the inclusion of female subjects, we now find a significant effect of Position on the absolute low‐ and high‐frequency response. In the supine position, LF and HF are on average 1.70 and 1.67 times higher than in the prone position (LF: *z* = 2.85, *p* = 0.004; HF: *z* = 2.166, *p* = 0.030), respectively.

**FIGURE 4:**
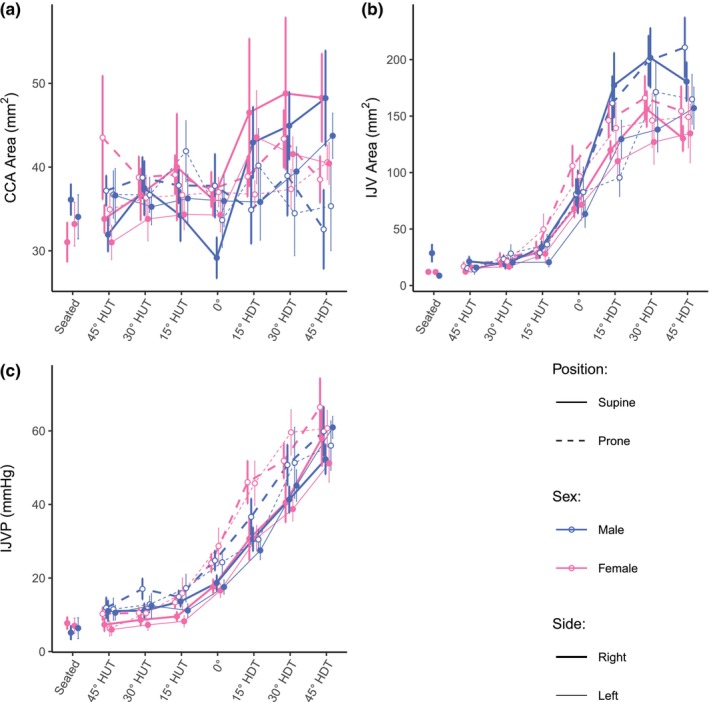
A_CCA_, A_IJV_, and IJVP as a function of tilt angle in supine and prone positions, in women and men. (a) A_CCA_, common carotid artery cross‐sectional area; (b) A_IJV_, internal jugular vein cross‐sectional area; (c) IJVP, internal jugular vein pressure. Measurements were collected at a seated baseline, 45° head‐up tilt (HUT), 30° HUT, 15° HUT, 0°, 15° head‐down tilt (HDT), 30° HDT, and 45° HDT. Data were collected on 12 male and 12 female participants. Data are presented as means ± SE at each tilt angle.

Figure 4 shows the evolution of A_CCA_, A_IJV_, and IJVP (mean ± SE) as a function of tilt angle (including the seated baseline). Table 3 reports the results of the GAMM analyses. With the addition of female subjects, we find no significant effect of Sex on any of the three neck variables (*p* = 0.557, *p* = 0.465, and *p* = 0.938 for A_CCA_, A_IJV_, and IJVP respectively). However, contrary to the male only results, we now find a significant effect of Side (*t* = −2.125, *p* = 0.034) and a small effect of tilt Angle (*F* = 1.659, *p* = 0.002 on the right side, *F* = 0.747, *p* = 0.021 on the left side) for A_CCA_. For A_IJV_ in male only subjects we saw a significant effect of *Side*, with the right IJV expanding more than the left. With the addition of female subjects, we now find an additional small significant effect of Position (*t* = 2.525, *p* = 0.012), such that A_IJV_ is slightly larger in the prone position compared to the supine position. For IJVP, the results are similar to those of male only subjects, in that we find no significant effect of Side (*t* = −0.469, *p* = 0.640) but do find a significant effect of Position (*t* = 6.653, *p* < 0.001), with IJVP being significantly higher in the prone position.

**TABLE 3:** Details of generalized additive mixed models (GAMM) analyses common carotid artery and internal jugular vein cross‐sectional area, and internal jugular vein pressure (IJVP).

	Parametric terms						Smooth terms^d^				Subject Std dev^g^ σ	Deviance explained^h^ %
	Position^a^		Side^b^		Sex^c^		$\sin\;\left(\text{Angle}\right)$ ^e^					
	t	p	t	p	t	p	Curve	EDF^f^	F	p		
$\sqrt{A_{\mathrm{CCA}}}$ ^i^ (mm)	−0.884	0.377	−2.125	0.034	0.587	0.557	Right	1.43	1.659	0.002	0.53	32.7
							Left	1.02	0.747	0.021		
$\sqrt{A_{\mathrm{IJV}}}$ ^i^ (mm)	2.525	0.012	−3.633	<0.001	−0.731	0.465	Supine/Right	4.21	75.865	<0.001	1.02	75.6
							Supine/Left	3.86	57.911	<0.001		
							Prone/Right	4.14	79.797	<0.001		
							Prone/Left	3.16	57.016	<0.001		
IJVP (mmHg)	6.653	<0.001	−0.469	0.640	0.078	0.938	Supine	3.69	107.743	<0.001	4.53	72.8
							Prone	3.79	133.329	<0.001		

*Note*: Models are fit to 12 male and 12 female participants. Significance of parametric and smoothed terms, effective degrees of freedom of smoothers, size of subject random effect, and model goodness of fit (deviance explained).

Abbreviations: A_CCA_, common carotid artery cross‐sectional area; A_IJV_, internal jugular vein cross‐sectional area; IJVP, internal jugular vein pressure.

^a^ Position: Supine or Prone; *t* reports effect size of prone compared to supine.

^b^ Side: Right or Left; *t* reports effect size of left compared to right.

^c^ Sex: Male or Female; *t* reports effect size of female compared to male.

^d^ Shrinkage‐penalized cubic regression splines fit to each significant position, side, and sex combination. Plots of the smoothers are included in the Appendix A1.

^e^ Sine of the tilt Angle in radians, positive values indicate head‐up tilt (HUT); *F* reports effect size.

^f^ Effective degrees of freedom.

^g^ Random effect $\gamma ∼N\left(0,\sigma^{2}\right)$.

^h^ Goodness of fit, equivalent to the unadjusted *R* ^2^.

^i^ See main text. Measurements of CCA and IJV size were homoscedastic in diameter, thus a square‐root transformation was used on the cross‐sectional area response.

### 3.2 Dose–response curves

**FIGURE 5:**
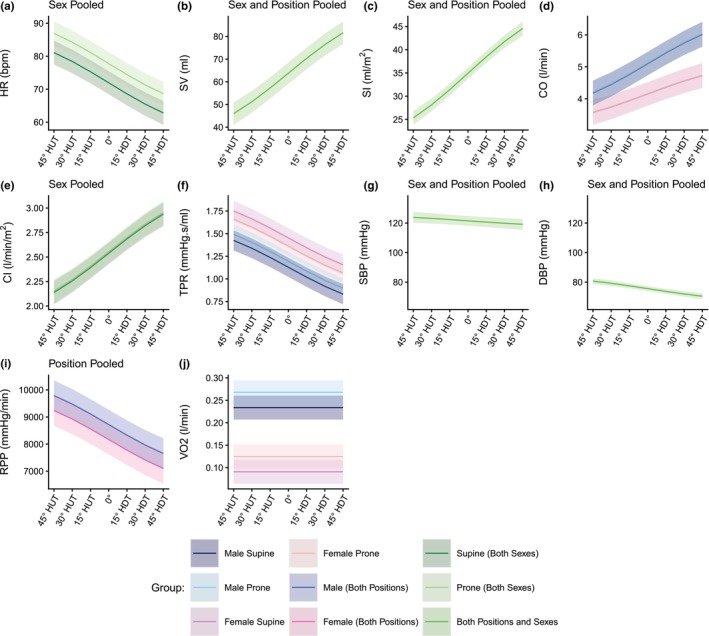
Estimated gravitational dose–response curves for hemodynamic parameters in the range 45° head‐up tilt (HUT) to 45° head‐down tilt (HDT) incorporating sex differences. (a) HR, heart rate; (b) SV, stroke volume; (c) SI, stroke volume index; (d) CO, cardiac output; (e) CI, cardiac index; (f) TPR, total peripheral resistance; (g) SBP, systolic blood pressure; (h) DBP, diastolic blood pressure; (i) RPP, rate pressure product; (j) V̇O_2_, oxygen consumption. Curves were fit via linear mixed‐effects models as described in the main text. Curves are presented as means ±95% confidence interval.

Figure 5 shows the estimated dose–response curves for the hemodynamic parameters considered within the range of 45° HUT to 45° HDT. The parameters for the dose–response curves are captured in Table 4. Curves are shown as mean and 95% confidence interval. Since there was no significant difference between male and female data for HR, SV, SI, CI, SBP, and DBP, sex data were pooled for those responses. Further, since there was no significant effect of *position* for SV, SI, SBP, DBP, and RPP, supine and prone estimates are also pooled for those dose response curves. Thus, for example, the dose–response curve for HR consists of two separate curves (supine and prone); the dose–response curve for RPP also consists of two curves (male and female), whereas the dose response curve for V̇O_2_ is four separate curves, one for each male/female and supine/prone combination. Similarly, the dose–response curves for SV, SBP, and DBP consist of a single curve for male/female and supine/prone data all pooled together.

**TABLE 4:** Estimated model coefficients for the gravitational dose–response curves displayed in Figures 5 and 6 generated by linear mixed models (LMMs) and generalized linear mixed models (GLMMs).

				Estimated coefficients^c^					Std dev of random effect^h^
	Model^a^	Link^b^	Units	$\beta_{0}$ intercept	$\beta_{1}$ $\sin\;\left(\text{Angle}\right)$ ^d^	$\beta_{2}$ sex^e^	$\beta_{3}$ position^f^	Additional β s^g^	
Hemodynamic measurements									
HR	LMM	μ=η	bpm	72.0 ± 1.8	12.9 ± 0.7	—	5.8 ± 0.7	—	8.4
SV	LMM	μ=η	mL	63.8 ± 2.4	−25.2 ± 1.2	—	—	—	11.3
SI	LMM	μ=η	mL/m^2^	34.9 ± 0.6	−13.6 ± 0.6	—	—	—	2.7
CO	LMM	μ=η	l/min	4.14 ± 0.19	−0.82 ± 0.09	0.95 ± 0.26	0.01 ± 0.06	Angle × Position: −0.48 ± 0.13	0.62
CI	LMM	μ=η	l/min/m^2^	2.54 ± 0.06	−0.57 ± 0.03	—	0.01 ± 0.03	—	0.24
TPR	LMM	μ=η	mmHg.s/mL	1.45 ± 0.05	0.42 ± 0.03	−0.32 ± 0.08	−0.09 ± 0.04	Position × Sex: 0.16 ± 0.05	0.16
SBP	LMM	μ=η	mmHg	121.5 ± 1.6	3.3 ± 1.3	—	—	—	7.5
DBP	LMM	μ=η	mmHg	75.6 ± 0.8	7.2 ± 0.9	—	—	—	3.5
RPP	LMM	μ=η	mmHg/min	8170 ± 280	1510 ± 100	550 ± 390	—	—	930
VO_2_	LMM	μ=η	l/min	0.091 ± 0.014	—	0.143 ± 0.019	0.034 ± 0.005	—	0.044
Time‐domain autonomic indices									
SDNN	GLMM	$\ln\left(\mu \right)=\eta$	ms	4.002 ± 0.055	−0.300 ± 0.029	—	−0.087 ± 0.028	—	0.253
RMSDD	GLMM	$\ln\left(\mu \right)=\eta$	ms	3.419 ± 0.083	−0.619 ± 0.038	—	—	—	0.396
HRVTi	GLMM	$\ln\left(\mu \right)=\eta$	—	2.484 ± 0.047	−0.149 ± 0.041	—	−0.062 ± 0.027	Angle × Position: −0.136 ± 0.058	0.210
BRS	GLMM	$\ln\left(\mu \right)=\eta$	ms/mmHg	2.521 ± 0.063	−0.684 ± 0.042	—	−0.154 ± 0.040	—	0.274
Frequency‐domain autonomic indices									
LF	GLMM	$\ln\left(\mu \right)=\eta$	ms^2^	6.665 ± 0.097	−0.338 ± 0.064	—	−0.281 ± 0.059	—	0.432
HF	GLMM	$\ln\left(\mu \right)=\eta$	ms^2^	5.653 ± 0.155	−1.141 ± 0.079	—	−0.134 ± 0.073	—	0.717
LFNorm	LMM	μ=η	—	70.5 ± 1.6	15.0 ± 1.2	—	—	—	7.3
HFNorm	LMM	μ=η	—	29.5 ± 1.6	−15.0 ± 1.2	—	—	—	7.3
LF/HF	GLMM	$\ln\left(\mu \right)=\eta$	—	0.899 ± 0.095	0.785 ± 0.085	0.302 ± 0.134	—	Angle × Sex: −0.159 ± 0.119	0.296

*Note*: Estimated coefficients are presented as mean ± SE. Only significant terms were included in the models.

^a^ All models use a linear predictor of the form: *η*_*ijk*_ = *β*_0_ + *β*_1_ sin (Angle) + *β*_2_ (Sex_*j*_) + β3 (Position_*k*_) + *β*_4_ (…) + *γ*_i_ + *ε*_*ijk*_ for subjects *i* (*i* = 1: 24), Sex *j* (*j* = 0: 1), and position *k* (*k* = 0: 1). All GLMMs have a Gamma distribution.

^b^ Link function between the linear predictor, *η*, and the expectation of the dependent variable, *μ*.

^c^ For GLMMs, coefficients *β* are given on the scale of the linear predictor for subject *i*, *η*_*i*_ = *Xβ* + γ_i_. The coefficient *β*_4_ corresponding to any significant interaction effects is noted where appropriate.

^d^ Sine of tilt angle from −0.707 (sin (−45°)) to 0.707 (sin (45°)), positive angles represent head‐up tilt, negative angles represent head‐down tilt.

^e^ Sex_*j*_: female = 0, male = 1.

^f^ Position_*k*_: supine = 0, prone = 1.

^g^ Additional coefficients for interaction effects as noted.

^h^ Standard deviation, *σ*, of random intercept, γ, for subject *i*. $\gamma_{i}∼N\left(0,\sigma^{2}\right)$. Units for *σ* are the same as the estimated coefficients.

**FIGURE 6:**
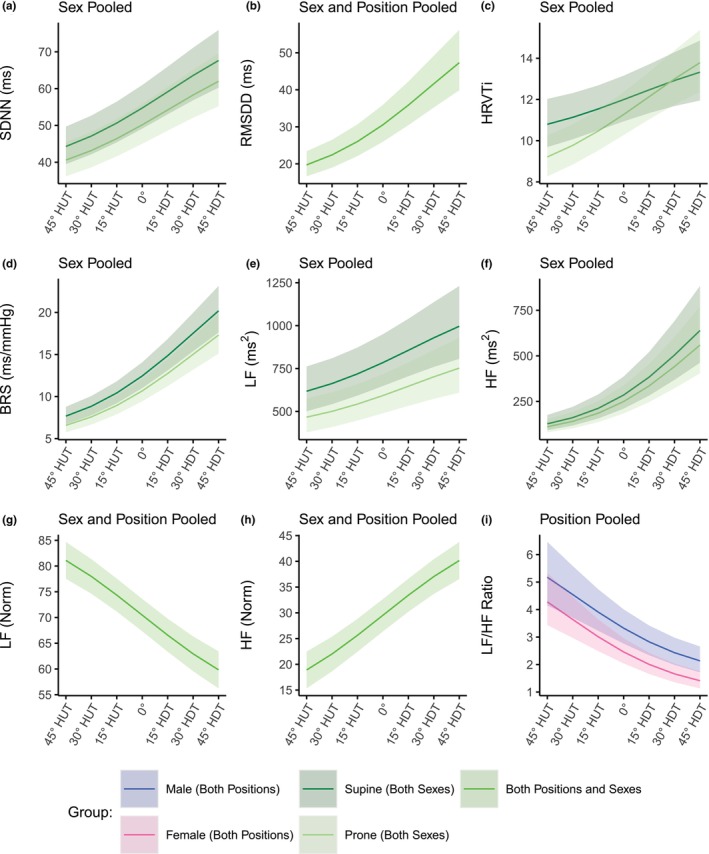
Estimated gravitational dose–response curves for autonomic parameters in the range 45° head‐up tilt (HUT) to 45° head‐down tilt (HDT) incorporating sex differences. (a) SDNN, standard deviation of NN intervals (normalized RR intervals); (b) RMSDD, root mean square of direct differences of NN intervals; (c) HRVTi, heart rate variability triangular index; (d) BRS, baroreceptor sensitivity; (e) LF, power density in the low frequency range (0.04–0.15 Hz); (f) HF, power density in the high frequency range (0.15–0.4 Hz); (g) LFNorm, LF (normalized units); (h) HFNorm, HF (normalized units); (i) LF/HF Ratio, ratio of low to high power densities. Curves were fit via linear mixed‐effects models (LFNorm, and HFNorm) and generalized linear mixed‐effects models (remaining parameters) as described in the main text. Curves are presented as means ± 95% confidence interval.

Figure 6 shows the estimated dose–response curves for the autonomic indices (both time domain and frequency‐domain) considered within the range of 45° HUT to 45° HDT. The parameters for the dose–response curves are captured in Table 4. Curves are shown as mean and 95% confidence interval. Since there was no significant difference between male and female data for any of the variables except LF/HF ratio, Sex data were pooled for those responses. Since there was no significant effect of Position for RMSDD, LF (Norm), HF (Norm), and LF/HF ratio, supine and prone estimates are also pooled for those dose–response curves.

**FIGURE 7:**
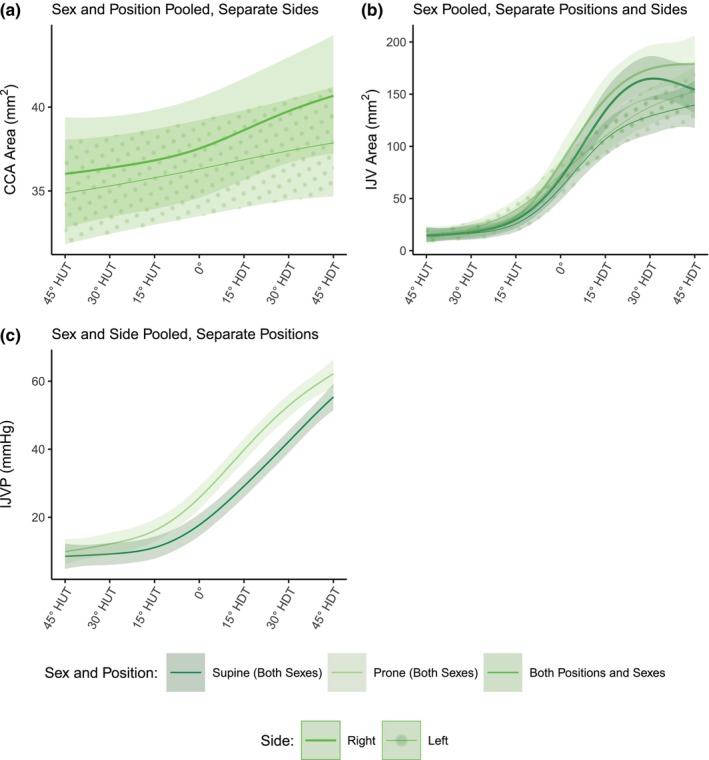
Estimated gravitational dose–response curves for ACCA, AIJV, and IJVP in the range 45° head‐up tilt (HUT) to 45° head‐down tilt (HDT) incorporating sex differences. (a) A_CCA_, common carotid artery cross‐sectional area; (b) A_IJV_, internal jugular vein cross‐sectional area; (c) IJVP, internal jugular vein pressure. Curves were fit via generalized additive mixed‐effects models as described in the main text. Curves are presented as means ± 95% confidence interval.

Figure 7 shows the estimated dose–response curves for A_CCA_, A_IJV_, and IJVP within the range of 45° HUT to 45° HDT. The effect sizes and model parameters are presented in Table 3. Curves are shown as mean and 95% confidence interval. Since there was no significant difference between male and female data for any of the variables, Sex data was pooled in all cases. Since there was no significant effect of Position for A_CCA_, supine and prone data were pooled. Finally, right and left side data were pooled for IJVP, since there was no significant effect of Side. As described previously (Whittle & Diaz‐Artiles, 2023), a square‐root transformation on the dependent variable was used to construct the dose–response curves for A_CCA_ and A_IJV_ since the data exhibited significant heterogeneity with regards to the area measurements as a function of tilt angle. The fitted smoothed terms used to construct the GAMMs in Figure 7 are presented in Appendix Figure S1.

## 4 DISCUSSION

This study augments our original work by adding female subjects and characterizing the effect of sex on cardiovascular hemodynamics and autonomic response. Our main findings show that: (1) most parameters measured do not exhibit a significant effect of sex, with significant differences only found in five out of 20 variables considered; (2) in the hemodynamic response, we find significant sex differences in CO, TPR, RPP, and V̇O_2_; (3) in the autonomic response, we only find significant sex differences in LF/HF ratio, a marker of sympathovagal balance; and (4) we find no sex effect in any of the variables related to carotid or jugular hemodynamics.

Considering the hemodynamic response, we only find a significant effect of sex in four of the measured variables. In particular, we find a sex effect in CO, TPR, RPP, and V̇O_2_, and we only find a significant interaction effect between sex and tilt angle in CO. When CO and SV were indexed to body surface area (CI and SI), the Sex by Angle effects disappeared, suggesting that the higher absolute cardiac output in men mainly reflects differences in body size. Diaz‐Canestro et al. hypothesized that sex differences in hemodynamic response to tilt can largely be explained by blood volume and oxygen carrying capacity (Diaz‐Canestro et al., 2022). Regarding the interaction effect, our results are congruent with Sarafian and Miles‐Chan, who found an interaction effect in CO between males and females in graded tilt, with males responding more strongly to tilt (Sarafian & Miles‐Chan, 2016). This is interesting and is potentially evidence of a different autonomic response between males and females. Badrov et al. found no sex differences in HR, SI, or CI, lending evidence to an anthropometrically driven difference. However, they also noted a significantly greater TPR in hypertensive female subjects in graded HUT (Badrov et al., 2020), which is matched in our study in normotensive subjects.

Indeed, although women tended to have slightly higher TPR, both sexes showed a very similar decrease in TPR in supine and prone positions. The modest Sex by Position interaction appeared to be influenced in part by variability at +45° HUT. We therefore interpret this effect as a subtle posture‐related modulation rather than a robust sex difference in peripheral vasoconstrictor responses.

Finally, Afrin Rimi et al. concluded that the cardiovascular response to tilting was less pronounced in females (Afrin Rimi et al., 2020). We observe this in the interaction effect of the CO response, but do not observe the significant interaction between sex and tilt angle for SBP that the authors noted. Finally, in our study, although absolute CO showed a significant Sex by Angle interaction (*p* = 0.011), the corresponding interaction for SV did not reach significance (*p* = 0.08), and HR did not exhibit any Sex or Sex by Angle effects. Taken together with the indexed analyses (CI and SI), this pattern suggests that the higher absolute CO in men is mostly explained by differences in body size and blood volume, with at most a modest contribution of sex‐specific SV responses.

We find marginal evidence of a differing autonomic response between males and females, given that we only found a significant effect of Sex in the LF/HF ratio, a marker of sympathovagal balance. However, LF power and LF/HF are known to reflect the combined influence of both sympathetic and parasympathetic inputs and are affected by factors such as respiration; thus, they should not be interpreted as direct measures of cardiac sympathetic activity alone. In this context, we interpret the LF/HF differences as reflecting shifts in overall sympathovagal balance rather than isolated sympathetic changes (Billman, 2013; Cooke et al., 2008; DeBeck et al., 2010; Eckberg, 1997). However, as with the male subjects, there is a large variance in the HRV metrics in female subjects, which could obscure smaller effect sizes of sex differences. Robertson et al. found that in upright tilt LF/HF increased more in males than in females (*p* = 0.044) (Robertson et al., 2020). They hypothesize that this is due to sympathetic modulation of HR to control blood pressure in men, versus more parasympathetic modulation in women. However, they also note a significant effect of sex on baroreflex sensitivity, which we do not observe. In a more chronic study, Schäfer Olstad et al. found that male runners exhibited higher markers of sympathetic activity during training and competitions, while females had higher markers of parasympathetic activity during training (Schäfer Olstad et al., 2017). Dart et al. seek to explain this difference by examining the effect of hormones on autonomic control (Dart et al., 2002). They note that estrogen enhances parasympathetic activity while promoting choline uptake and acetylcholine synthesis and release (Joyner et al., 2015). Conversely, they present evidence that testosterone enhances norepinephrine (NE) and neuropeptide Y (NPY) synthesis and reduces NE clearance (Baker et al., 1978; Zukowska‐Grojec, 1995; Zukowska‐Grojec et al., 1991). Both of these are sympathetic co‐transmitters, lending support to greater sympathetic activity in males and greater parasympathetic activity in females.

Finally, we do not find any sex‐dependent differences in A_CCA_, A_IJV_, or IJVP. This is in contrast to Patterson et al., who noted greater jugular venous attenuation in males than in females in HDT (Patterson et al., 2022). However, in a study measuring the influence of a neck compression collar on cerebrovascular and autonomic function, Joshi et al. found no sex effect in A_CCA_ or A_IJV_ in the baseline condition (Joshi et al., 2019). However, the authors did find a significant effect of sex when wearing the compression collar on A_IJV_ at end inhalation and on both A_CCA_ and A_IJV_ at end‐exhalation. They hypothesize that these differences are likely explained by the previously identified autonomic differences between males and females. We could find no studies examining sex differences in IJVP between males and females; however, studies of central venous pressure found no significant difference between males and females (Convertino, 1998). In light of recent reports of jugular venous engorgement, stagnant or retrograde IJV flow, and in‐flight IJV thrombosis (Auñón‐Chancellor et al., 2020; Lee et al., 2020; Marshall‐Goebel et al., 2019), the combined assessment of A_CCA_ and A_IJV_ cross‐sectional areas together with IJVP in the present study offers a focused description of how acute changes in gravitational loading alter neck vascular volume and pressure. These neck vascular responses help to contextualize cephalic fluid shifts in spaceflight and ground‐based analogs, whereas more detailed intracranial hemodynamics need to be studied in further works.

### 4.1 Implication for countermeasure design

These findings have important implications for both spaceflight health management and the design of countermeasures targeting cardiovascular deconditioning. As the astronaut population becomes more diverse, including increasing numbers of women, understanding sex‐specific cardiovascular responses to gravitational changes is critical for optimizing pre‐flight screening, in‐flight countermeasures, and post‐flight rehabilitation protocols. While our results regarding the gravitational dependence of central hemodynamics and vascular morphology appear largely consistent across sexes, specific differences in cardiac output and autonomic balance may influence individual susceptibility to orthostatic intolerance following spaceflight. Additionally, our observation of sex‐independent responses in carotid and jugular variables suggests that venous flow patterns and vessel morphology (critical factors in thrombosis risk) are not significantly influenced by sex under these acute altered gravity conditions.

As shown in Figures 5, 6, 7, the estimated dose–response curves for hemodynamic, autonomic, and carotid/jugular characteristics derived from large tilt angle variations allow projecting gravitational vector components along the craniocaudal axis. A gravity load equivalent to different environments can then be estimated: 0 G and −6° head‐down tilt for spaceflight, 0.16 G and +9.2° head‐up tilt for Moon gravity, and 0.38 G and +22.3° head‐up tilt for Mars gravity. These lunar and Martian gravity equivalent levels are encompassed by our tilt range.

Thus, our study provides a comprehensive cardiovascular dose–response map across a range of gravitational loading, from head‐down to head‐up tilt, in which gravitational stress and vascular responses are altered. While the present protocol focuses on acute responses, the graded tilt dose–response profiles we report offer a useful framework for interpreting findings from longer‐duration head‐down tilt and other spaceflight analogs, and for refining sex‐specific hypotheses and countermeasure strategies. Specifically, our findings may guide the adjustment of physical exercise regimens (load, duration, frequency) (DeVirgiliis, 2025; Fernandez‐Gonzalo et al., 2025; Scott et al., 2023), fluid loading strategies (volume and intensity) (Fu et al., 2019; Kurazumi et al., 2022), and compression garment protocols (pressure load, duration of use) (Lee et al., 2025; Reinarz, 2025). Beyond space applications, these insights may also inform clinical practices related to syncope, Trendelenburg surgery, or rehabilitation in bedridden or elderly patients, where gravitational stress and vascular responses are altered.

As research perspectives, further studies may focus on comparing different altered gravity paradigms in both men and women (e.g., tilt test, lower body negative pressure [LBNP], centrifuge (Diaz‐Artiles et al., 2018)) to explore the comprehensive cardiovascular responses to changes in gravitational vector. It is critical to also include the ophthalmic side, since spaceflight‐induced neuro‐ocular syndrome (SANS) has been found in astronauts. Intraocular pressure has already been shown to be dependent of the gravitational vector in tilt and LBNP paradigms without showing sex differences, consistently with our findings here (Hall et al., 2024; Petersen et al., 2022; Whittle et al., 2025).

### 4.2 Limitations

The limitations for this experiment remain broadly as described in Whittle & Diaz‐Artiles (2023) (progressive tilt instead of randomized, only noninvasive measurement of IJVP, no flow measurement). Here, the male and female cohorts were acquired with different handheld scanners for vascular B‐mode imaging (VScan Extend vs. Butterfly iQ+) since data collection occurred at a different time for both groups. However, we used the same approach to localize and capture the cross‐sectional area image (measurements collected 3 cm inferior to the CCA bifurcation point, around the C3 vertebral level). Moreover, contemporary handheld systems (including Butterfly iQ+) have demonstrated acceptable construct validity and reproducibility for carotid diameter compared with high‐end cart‐based ultrasound, and handheld ultrasound devices show comparable diagnostic accuracy across cardiovascular applications (Gibbons et al., 2024; Jin et al., 2022; Willems et al., 2023). As such, we do not believe that this measurement difference invalidates any conclusions.

In addition, in female participants we did not control for menstrual cycle phase or hormonal contraceptive use. Although hormonal fluctuations may influence cardiovascular and autonomic regulation, the present protocol examined acute responses to a strong graded orthostatic stimulus, for which the primary effects are likely driven by changes in gravitational stress.

Finally, although we report LF, HF, and LF/HF for HRV, these indices are influenced by both sympathetic and parasympathetic inputs and should be interpreted as markers of overall autonomic modulation rather than direct measures of cardiac sympathetic activity.

## 5 CONCLUSION

We augmented our initial experiment by incorporating female subjects in order to characterize the effects of sex on cardiovascular parameters in graded tilt. Our data revealed that only a few variables displayed a significant effect of sex. In particular, we found a significant effect of sex in CO, TPR, RPP, V̇O_2_, and LF/HF ratio. Further, we only found a significant interaction effect between sex and tilt angle in two variables: CO and LF/HF ratio. The greater increase in CO seen in male subjects with increasing HDT is likely due to the larger blood volume in males. Overall, the data reveal that there are not large sex differences in the hemodynamic response to tilt. The dose–response curves generated here can also support the validation of computational models to predict individual cardiovascular response to altered gravitational environment (Diaz‐Artiles et al., 2019; Fois et al., 2024; Whittle & Diaz‐Artiles, 2021). Finally, these findings augment our original male‐only experiment and now inform the development of more personalized spaceflight countermeasures for crewmembers of both sexes.

## AUTHOR CONTRIBUTIONS

Richard S. Whittle and Ana Diaz‐Artiles contributed to the study conception and design. Material preparation, data collection, analysis, and interpretation were performed by Richard S. Whittle, Adrien Robin, and Ana Diaz‐Artiles. The first draft of the manuscript was written by Adrien Robin and Richard S. Whittle and all authors commented on previous versions of the manuscript. All authors read and approved the final manuscript.

## FUNDING INFORMATION

This work was supported by the National Aeronautics and Space Administration (NASA) Human Research Program (HRP), Grant 80NSSC20K1521 and by the Translational Research Institute for Space Health through NASA Cooperative Agreement NNX16AO69A.

## CONFLICT OF INTEREST STATEMENT

The authors have no relevant financial or non‐financial interests to disclose.

## Supporting information


Data S1.

Data S2.

## Data Availability

The datasets analyzed for this study are publicly available, a repository can be found on GitHub: https://github.com/BHP‐Lab/tilt‐dose‐response.
