# Supplementary material for: Cardiovascular response to altered gravity in healthy adults: Insight from graded tilt testing
Source: Physiol Rep. 2026 Feb 19;14(4):e70782. doi: 10.14814/phy2.70782 (PMC12920070; doi:10.14814/phy2.70782)
Supplement: Supplementary file 1 — Data S1. [file PHY2-14-e70782-s002.docx]

**APPENDIX**

**Figure S1**: Fitted smoothed terms for generalized additive mixed-effects models (GAMMs) incorporating sex differences.
