# Supplementary figures and images for: Cardiovascular response to altered gravity in healthy adults: Insight from graded tilt testing
Source: Physiol Rep. 2026 Feb 19;14(4):e70782. doi: 10.14814/phy2.70782 (PMC12920070; doi:10.14814/phy2.70782)

### $\sqrt{A_{CCA}}$ Smoothers

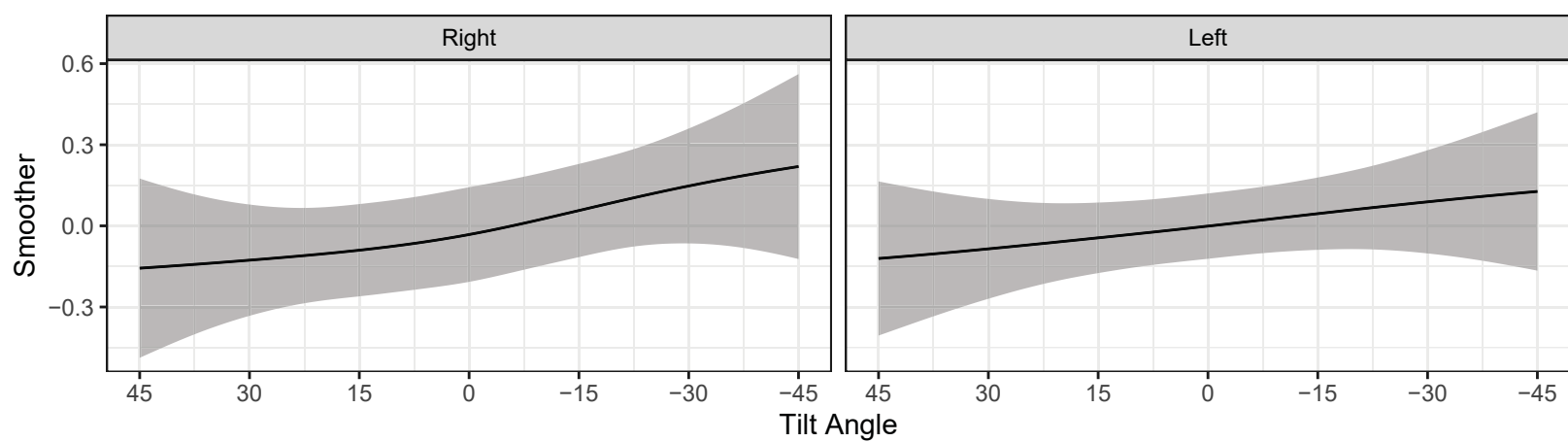

### $\sqrt{A_{IJV}}$ Smoothers

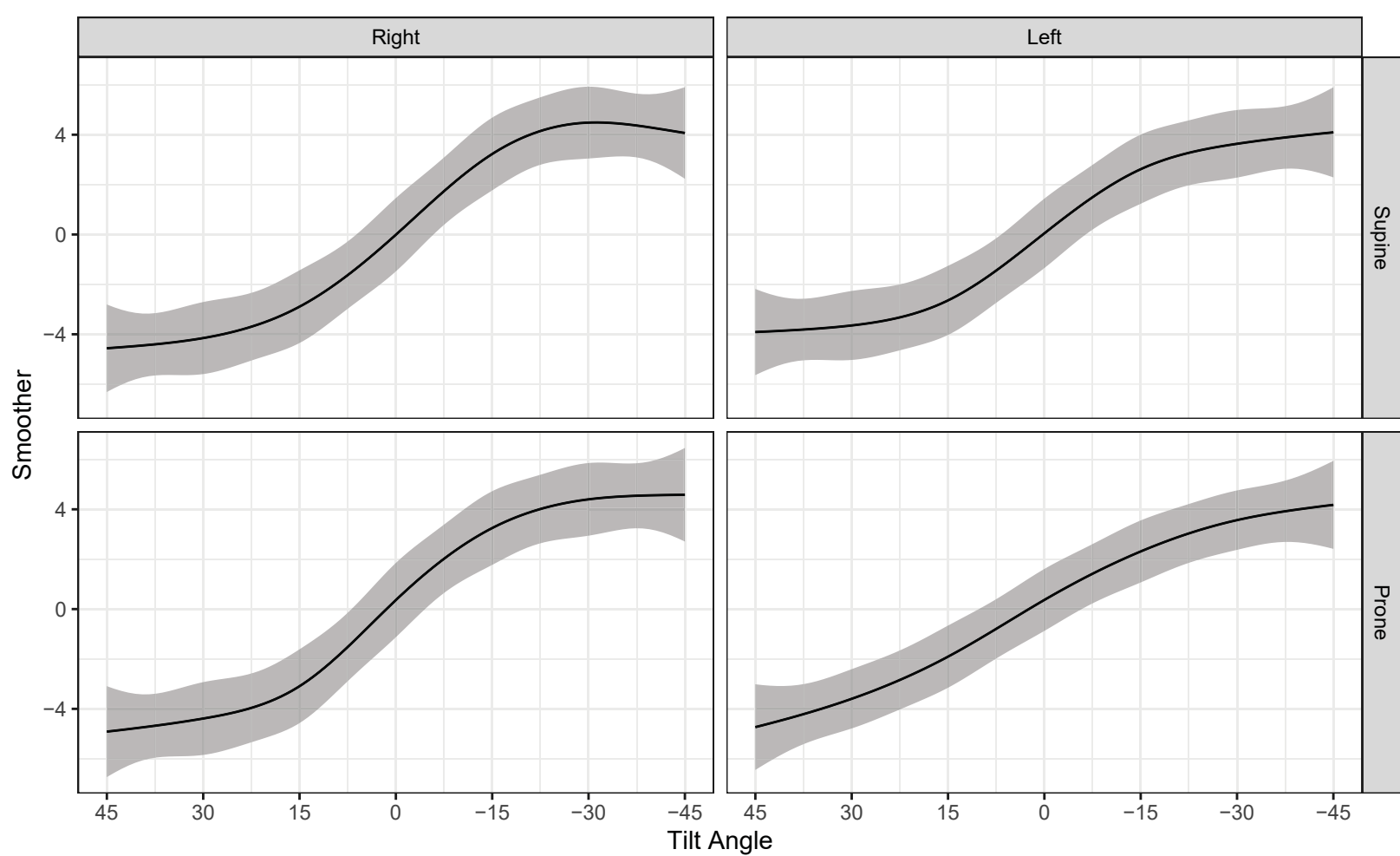

### IJVP Smoothers

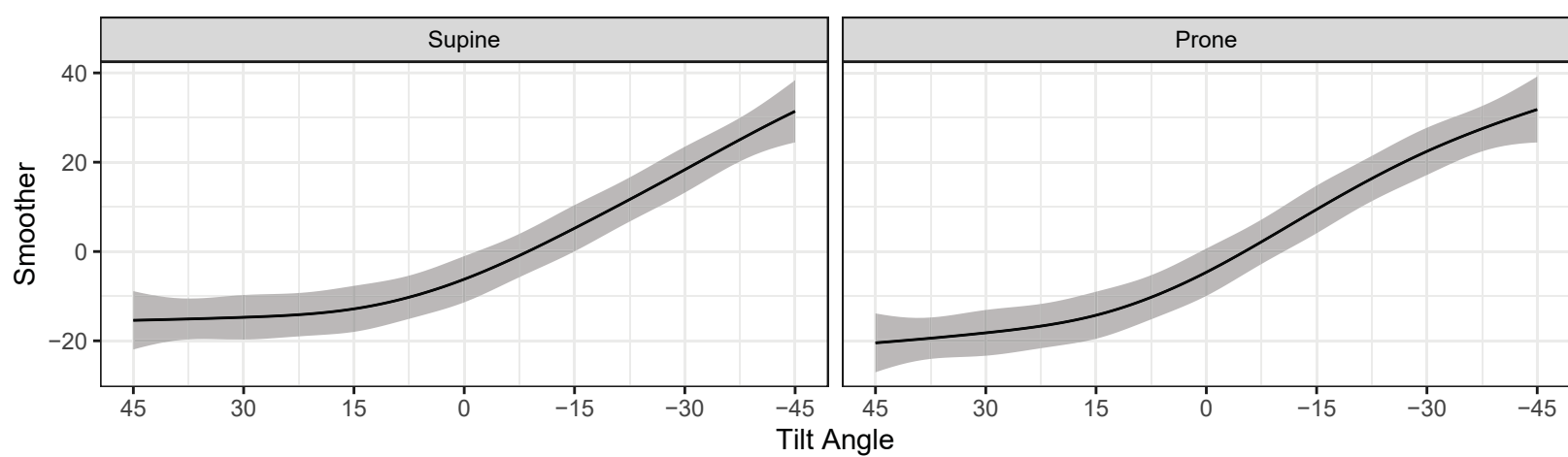

Supplement: Supplementary file 2 — Data S2. [file PHY2-14-e70782-s001.pdf]
